# Improvement of Micro-Hole Processing in SiC_f_/SiC Ceramic Matrix Composite Using Efficient Two-Step Laser Drilling

**DOI:** 10.3390/mi16040430

**Published:** 2025-04-02

**Authors:** Yue Cao, Bin Wang, Zhehang Li, Jiajia Wang, Yinan Xiao, Qingyang Zeng, Xinfeng Wang, Wenwu Zhang, Qunli Zhang, Liyuan Sheng

**Affiliations:** 1 College of Mechanical Engineering, Zhejiang University of Technology, Hangzhou 310014, China; caoyue@nimte.ac.cn (Y.C.); lizhehang@nimte.ac.cn (Z.L.); wangxinfeng@nimte.ac.cn (X.W.); zql@zjut.edu.cn (Q.Z.); 2 Ningbo Institute of Materials Technology and Engineering, Chinese Academy of Sciences, Ningbo 315201, China; wangjiajia@nimte.ac.cn (J.W.); zengqingyang@nimte.ac.cn (Q.Z.); zhangwenwu@nimte.ac.cn (W.Z.); 3 PKU-HKUST Shenzhen-Hongkong Institution, Shenzhen 518057, China; ynxiao_pkusz@yeah.net; 4 Shenzhen Institute, Peking University, Shenzhen 518057, China

**Keywords:** SiC_f_/SiC ceramic matrix composite, two-step laser drilling, micro-hole, laser ablation mechanism

## Abstract

SiC_f_/SiC ceramic matrix composite (CMC), a hard and brittle material, faces significant challenges in efficient and high-quality processing of small-sized shapes. To address these challenges, the nanosecond laser was used to process micro-holes in the SiC_f_/SiC CMC using a two-step drilling method, including laser pre-drilling in air and laser final-drilling with a water jet. The results of the single-parameter variation and optimized orthogonal experiments reveal that the optimal parameters for laser pre-drilling in air to process micro-holes are as follows: 1000 processing cycles, 0.7 mJ single-pulse energy, −4 mm defocus, 15 kHz pulse-repetition frequency, and 85% overlap rate. With these settings, a micro-hole with an entrance diameter of 343 μm and a taper angle of 1.19° can be processed in 100 s, demonstrating high processing efficiency. However, the entrance region exhibits spattering slags with oxidation, while the sidewall is covered by the recast layer with a wrinkled morphology and attached oxides. These effects are primarily attributed to the presence of oxygen, which enhances processing efficiency but promotes oxidation. For the laser final-drilling with a water jet, the balanced parameters for micro-hole processing are as follows: 2000 processing cycles, 0.6 mJ single-pulse energy, −4 mm defocus, 10 kHz pulse-repetition frequency, 85% overlap rate, and a 4.03 m/s water jet velocity. Using these parameters, the pre-drilled micro-hole can be finally processed in 96 s, yielding an entrance diameter of 423 μm and a taper angle of 0.36°. Due to the effective elimination of spattering slags and oxides by the water jet, the final micro-hole exhibits a clean sidewall with microgrooves, indicating high-quality micro-hole processing. The sidewall morphology could be ascribed to the different physical properties of SiC fiber and matrix, with steam explosion and cavitation erosion. This two-step laser drilling may provide new insights into the high-quality and efficient processing of SiC_f_/SiC CMC with small-sized holes.

## 1. Introduction

In the aerospace industry, advanced materials have played important roles in different aspects, such as aero-engine, aerofoil, braking system, etc. [1,2,3]. The applications of lightweight materials in airframe and ultrahigh-temperature materials in aero-engines increase fuel efficiency and decrease greenhouse gas emissions, which promotes innovation in the aerospace industry [4,5]. Especially for the aero-engine, its increased thrust-to-weight ratio results in a rise in the working temperature, which increases the requirement of hot-end components. Among them, the combustor chamber and turbine blade have to endure a high-temperature, high-pressure, and high-speed airflow [6,7]. Therefore, their materials should possess a high melting point, balanced fatigue properties, excellent oxidation resistance, and high-temperature strength. In fact, the widely used superalloys have not completely achieved their maximum performance because of their melting point limitations [8,9,10]. Thus, it is necessary to develop new materials with excellent thermal and mechanical properties, meeting these strict requirements. Among numerous materials, SiC fiber-reinforced/SiC (SiC_f_/SiC) ceramic matrix composite (CMC) has been recognized as one of the most promising thermal structural materials in aero-engines and nuclear reactors, due to its ultrahigh melting temperature, high thermal stability, high specific modulus, low density, excellent high-temperature strength, and outstanding wear resistance [11,12,13]. As a combustor material, the SiC_f_/SiC CMC has to machine a large number of air film cooling holes, achieving its effective cooling and temperature reduction. However, this brings many challenges in machining because of the intrinsic brittleness of SiC and its high stiffness [14,15]. On the contrary, the combustor has many requirements for machined holes, such as high precision, low damage, small thermal affected zone, and high machining efficiency [16]. It becomes a great challenge to balance these requirements. The intrinsic features of SiC, the reinforced SiC fibers, further increase the machining difficulty of the SiC_f_/SiC CMC. Due to the ultrahigh strength of SiC fiber along the axial direction, the core of the SiC_f_/SiC CMC has high toughness, but its relatively low radial strength always becomes a reason for failure [17,18]. Once the SiC fiber is broken, the strengthening effect decreases significantly.

Thus, the integrity of the SiC fiber is so important that it determines the performance and service life of the SiC_f_/SiC CMC. However, the removal mechanism of conventional machining methods, such as milling and drilling, mainly depends on the loading force and scraping [19]. Due to the high difference in strength between the SiC fiber and SiC matrix, high stress is generated along the SiC fiber and the SiC matrix interface during conventional machining, which leads to interfacial cracking [20]. The ultrasonic-assisted machining could improve the processing quality and efficiency, but the wear rates of the tools are increased greatly, which increases the difficulty of quality control and the corresponding cost [21]. The electrical discharge machining (EDM) may achieve the high-precision processing of SiC_f_/SiC CMC with an obviously decreased cracking along its interface, but the instantaneous exposure to high temperature caused by the electrical discharge produces the distinct recast layer and thermal affected zone, which decrease the interfacial stability [22]. The combination of mechanical machining and EDM may enhance efficiency and quality, but the high machining stress also results in defects, such as debonding and microcracks, along the fiber and matrix [23]. Moreover, the low electrical conductivity of SiC phase also increases the difficulty of machining and decreases the machining efficiency [24]. The abrasive water jet machining may effectively solve the issue of the formed recast layer and thermal affected zone in the process, but its high jet impact force may damage the outlet position [25,26]. Additionally, the abrasive water jet machining is only adapted to the processing of simple shapes, and its processing precision is limited, which hinders its application. Thus, it is crucial to explore a new method and procedure to process the SiC_f_/SiC CMC with satisfactory quality and efficiency.

Recently, laser processing has attracted much attention because of its non-contacting processing mode, high precision, high energy density, flexible assembly, etc. [27,28,29]. Compared with the conventional machining method, laser processing could obtain a processed surface with few recast layers and low interfacial stress. In addition, the processing efficiency and quality of laser processing could be well regulated by optimizing parameters [30,31]. Furthermore, the influence of tool wear on the precision of processed structures need not be considered. However, the difference in fiber and matrix still influences the laser processing and ablated surface. Jiao et al. [32] utilized the nanosecond laser to fabricate blind holes in the 2.5-dimensional carbon fiber-reinforced SiC ceramic composite and revealed that the depth of blind holes is linearly related to the scanning number when the focus displacement is set. Wu et al. [33] applied a laser with a wavelength of 1064 nm to process the unidirectional C_f_/SiC composite and demonstrated that the fiber orientation greatly affected the ablated surface morphology. The formed ridge always became the origin of crack, due to the oxidation of carbon fibers during laser ablation. The research on femtosecond laser processing SiC with different pulse repetition rates and assisting mediums exhibited that the assisting medium could play an important role during the drilling [34]. The laser machining of SiC-based composite is mainly achieved via the vaporization and removal of melted debris. It implies that the diverse physical properties of fibers and matrices affect the machining consistency.

Though the SiC fiber-reinforced/SiC CMC has almost the same chemical content, there are still some differences pertaining to density and thermal transfer behavior [35,36]. Such subtle differences influence the local ablation, thermal affected zone, and interface stress. Therefore, the laser processing parameters, such as instantaneous input energy, pulse frequency, and assisting medium, would play an important role in the machining of SiC_f_/SiC CMC. Rao et al. [37] applied the rotational thirteen-beam coupling nanosecond laser to drill SiC_f_/SiC CMC and obtained small-sized holes, which demonstrated that the change in rotational speed could adjust the shape of holes and their surface morphology. Zhang et al. [38] utilized a picosecond laser to machine the microgrooves in SiC_f_/SiC CMC, which revealed that the high input energy, high scanning time, and low scanning speed were beneficial to machining efficiency but detrimental to surface roughness. Moreover, the amorphous SiO_2_ formed on the machined surface or as debris. Yang et al. [39] used the femtosecond laser with a rotary beam to drill SiC_f_/SiC CMC and exhibited that the high pulse and rotary speed effectively improved the hole aspect and decreased the hole taper. Moreover, the thermal damage on the machined surface was significantly decreased. Though the enhanced laser pulse helps the machining precision, the machining efficiency declines simultaneously. Recent studies [27,40] have utilized water as a cooling medium to decrease the surface oxides and thermal affected zone, which could increase the surface quality but contribute little to machining efficiency. Xu et al. [41] applied a UV laser to cut thick pieces of SiC_f_/SiC CMC in air and demonstrated that the multiline layered scanning method increased the cutting depth and aspect ratio, while the presence of oxygen promoted the oxidation reaction. It can be seen that the regulation of a single factor for laser machining could not simultaneously improve the efficiency and quality. Recent research on laser-processed superalloys has revealed that a femtosecond laser with a two-step spiral drilling method can obtain high-quality micro-holes with high efficiency. Then, it can be deduced that the multi-step laser machining with an appropriate assisting medium may achieve the machining of SiC_f_/SiC CMC with high efficiency and quality. Madhukar et al. [42] found that coaxial waterjet-assisted fiber laser processing has significant advantages over gas-assisted processing, especially in cutting. However, research on the waterjet-assisted laser processing of CMCs is still limited, and studies on this combined method remain scarce. Thus, it is worthwhile to explore the related mechanism and optimal parameters.

In this study, two-step laser processing was designed and applied to machine micro-holes in SiC_f_/SiC CMC, which included the pre-drilling step of gas-assisted laser processing and the final-drilling step of coaxial water jet-assisted laser processing. The pre-drilling had relatively high machining efficiency, while the final-drilling had relatively high precision. The orthogonal experiments were performed to explore the optimum laser drilling procedure for SiC_f_/SiC CMC. The micro-hole shape, surface morphology, and chemical composition of the machined SiC_f_/SiC CMC were characterized to reveal the material removal mechanism. It is expected that two-step laser processing could improve the machining efficiency and quality of the CMCs.

## 2. Material and Experiment

### 2.1. Material

**Figure 1:**
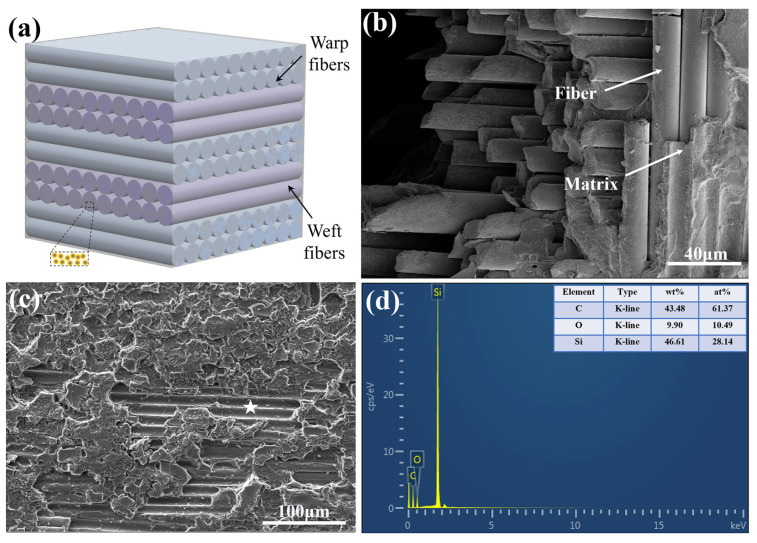
Structure and morphology of SiC_f_/SiC CMC: (**a**) three-dimensional schematic diagram of SiC_f_/SiC CMC; (**b**) microstructure of cross-section of SiC_f_/SiC CMC; (**c**) surface morphology of SiC_f_/SiC CMC; (**d**) EDS analysis of region with exfoliated SiC fiber marked with star.

The SiC_f_/SiC CMC used in this experiment was manufactured through the chemical vapor infiltration (CVI) process [12]. For the SiC_f_/SiC CMC fabricated by the CVI process, a relatively intact fiber weave layer was obtained only during component fabrication. However, this process would result in the high porosity in the infiltration layer or interface. As shown in Figure 1a,b, the used SiC_f_/SiC CMC mainly comprised intersected SiC fibers, arranging in a 0°/90° alternating weave pattern and stack. The diameter of SiC fiber is approximately 12 μm, and the gaps among SiC fibers were filled by the deposited SiC matrix. The observation on the surface of the SiC_f_/SiC CMC exhibited the exfoliated SiC fibers, and the EDS test revealed the presence of a relatively high oxygen content, as shown in Figure 1c,d. The thickness of used SiC_f_/SiC CMC sheet was 3 mm, and all composite sheets were ultrasonically cleaned with ethyl alcohol before laser processing.

### 2.2. Laser Drilling

**Figure 2:**
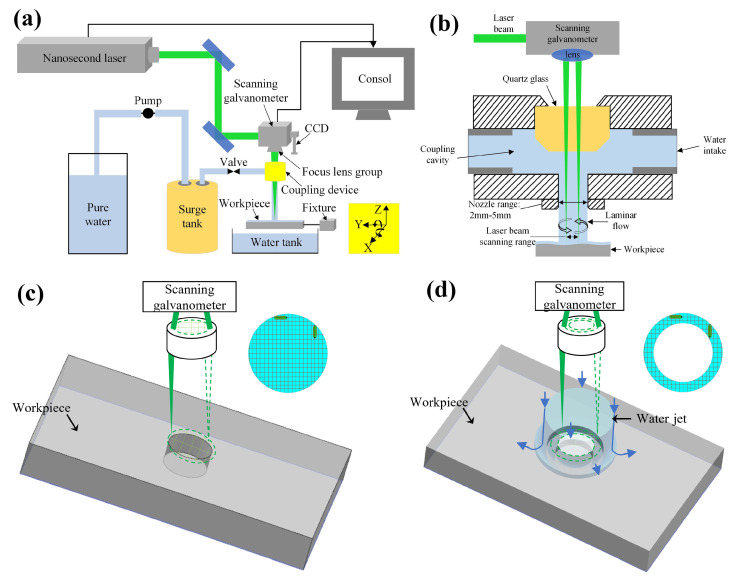
Schematic diagram of laser-processed SiC_f_/SiC CMC and detailed scanning path for micro-hole: (**a**) the laser processing platform; (**b**) laser–waterjet coupling device; (**c**) scanning path of laser pre-drilling in air; (**d**) scanning path of laser final-drilling with water jet.

The objective of this study was to drill micro-holes with diameters of less than 600 µm in the SiC_f_/SiC CMC, by which the optimal two-step laser drilling for this kind of composite could be obtained. To achieve this aim, the SiC_f_/SiC CMC was drilled using the laser processing platform developed by our research group. This laser processing platform was mainly composed of the five-axis motion system, controlling system, nanosecond laser, scanning mirrors, CCD, and optical transmission system, as shown in Figure 2a. The solid-state Nd: YAG laser (Edge Wave Inno Slab IS8I-E, Würselen, Germany) was used to generate a nanosecond pulsed laser with a wavelength of 532 nm, a pulse width of 10.9 ns, a pulse frequency of 1–50 kHz, and a pulse energy of 0.2–0.7 mJ. With the laser processing platform, the parameters, such as overlap rate, pulse energy, defocus, and pulse-repetition frequency, could be regulated to achieve a reasonable procedure. The coaxial water jet-assisted system developed by our research group was employed to achieve high-precision laser processing. During the laser processing, the coaxial water jet-assisted system was fixed to the scanning galvanometer to ensure the consistency of the processed position. When the laser pre-drilling was performed on the laser processing platform, the water jet valve was closed. Otherwise, it was opened to enable the coupling of laser and water. The water jet was coupled with the laser beam through the outlet, and the water jet speed could be regulated by a valve, as shown in Figure 2b. With the help of the water jet, the laser-processed surface could be cooled effectively, and the formed debris could be taken away rapidly.

To achieve high efficiency, the laser pre-drilling was performed on the SiC_f_/SiC CMC in air, which could make full use of the input energy and accelerate the ablation. Though the high input energy and low scanning speed could increase the machining efficiency, they also resulted in a thick oxide layer and a bigger thermal affected zone, which increased the difficulty of subsequent laser finishing. To obtain the balanced laser processing effect, the typical experiments with a single changed factor, such as processing time, spot overlap rate, single-pulse energy, defocus amount, and pulse-repetition frequency, were performed. The micro-holes were prepared by laser processing with the scanning fill method, as shown in Figure 2c. The laser scanning route of pre-drilling was controlled by a galvanometer to achieve movement along the X and Y axes. The optimal parameters were determined by the orthogonal experiments. The laser final-drilling with a water jet was performed on the drilled SiC_f_/SiC CMC to remove the surface layer of the micro-holes’ inner wall, which improved the surface roughness and hole shape. This processing was achieved by the concentric circular scanning filling method, as shown in Figure 2d. Therefore, the laser scanning route of the laser final-drilling with a water jet was of a ring shape. In addition to the laser parameters, the water jet velocity could influence the laser processing effect, because it determines the cooling rate and the thickness of the thermal affected zone. Thus, the typical experiments with a single changed factor, such as water jet velocity, processing time, spot overlap rate, single-pulse energy, defocus amount, and pulse-repetition frequency, were also performed.

During the setting of laser processing parameters, there are some important parameters that had a clear influencing tendency. Generally, the increase in processing time, overlap rate, and pulse energy enhanced the absorbed heat density of processed region in an environment without a special medium, which contributed to ablation but increased the thermal affected zone and affected surface morphology. The defocus had a two-sided influence; the higher the defocus, the lower the heat density. The overfocus and underfocus both decreased the absorbed heat density of processed region, but the processing area was increased. The increase in pulse-repetition frequency decreased the input energy density. The increase in water jet velocity removed more heat, which decreased the thermal affected zone and benefited the processing precision. Based on these basic rules, the detailed laser processing parameters were set for laser pre-drilling in air and laser final-drilling with a water jet

#### 2.2.1. Laser Pre-Drilling in Air

Based on the previous studies [43,44], the basic parameters of laser pre-drilling in air were set as follows: 900 processing cycles, 80% overlap rate, 0.5 mJ single-pulse energy, −1 mm defocus, and 20 kHz pulse-repetition frequency. When one parameter was changed, the other parameters were set as basic values. The varied parameters for single-parameter experiments of laser pre-drilling in air are shown in Table 1. For drilled micro-holes, the primary requirement is the hole shape, and the taper angle is the determining factor. To identify the optimal parameters, laser pre-drilling was performed three times for each parameter group to obtain the average taper angle. Based on the results of the single-factor experiments, the parameters with the most significant influence were selected and combined for further study.

#### 2.2.2. Laser Final-Drilling with Water Jet

For the laser final-drilling with a water jet, its parameter changing range was narrowed because of the excellent cooling effect of the water jet. The specimen for laser final-drilling with a water jet was the SiC_f_/SiC CMC with an optimal pre-drilled micro-hole. Considering the cooling effect of the water jet, the basic parameters of laser final-drilling with a water jet were set as follows: 2000 processing cycles, 80% overlap rate, 0.6 mJ single-pulse energy, 0 mm defocus, 20 kHz pulse-repetition frequency, and 5.43 m/s waterjet velocity. The varied parameters for single-parameter experiments of laser final-drilling with a water jet are shown in Table 2. The water jet velocity was a crucial factor in this section. The diameter of the coaxial water jet device nozzle was 2 mm. The water jet velocity was calculated using Formula (1), where *V* is the volume of water discharged in time *t*, and *d* is the diameter of the nozzle [45].

(1) $$v=\frac{4V}{\pi d^{2}t}$$

The final laser drilling was performed three times for each parameter group to obtain the average taper angle. Based on the results of the single-factor experiments, the parameters with significant influence were selected and combined for further investigation.

### 2.3. Characterization

The laser-processed SiC_f_/SiC CMC specimens were ultrasonically cleaned with anhydrous ethanol for 15 min to remove any attached debris. The shapes and dimensions of the laser-processed micro-holes were characterized using a laser scanning confocal microscope (LSCM, VK-X200K, Osaka, Japan). During the analysis of micro-holes in the SiC_f_/SiC CMC, the microscope slides were used as the supporter. The surface morphology and elemental distribution of the micro-holes, with varying processing conditions, were observed and analyzed using scanning electron microscopy (SEM, FEI Quanta FEG 250, FEI, Hillsboro, OR, USA) coupled with an energy-dispersive spectrometer (EDS, Oxford X-MAX 50, Oxford Instruments, Oxford, UK). To examine the detailed variations in surface chemical bonds, X-ray photoelectron spectroscopy (XPS, AXIS SUPRA, Kratos, Manchester, UK) was employed to analyze the laser-processed composite surface.

During laser drilling, the continuous irradiation of SiCf/SiC CMC resulted in high temperatures, which induce thermal expansion. Comparatively, the bottom absorbed more heat and experienced a higher temperature, which induced a gradual thermal expansion. Consequently, it affected the dimensions of the laser-processed hole from surface to bottom, resulting in a tapered shape. In fact, the tapered shape influenced the subsequent application, and the taper angle was set as the main factor. The taper angle is calculated using the following formula [46]:

(2) $$\theta =arctan\frac{D_{1}-D_{2}}{2h}\times \frac{180{}^{\circ}}{\pi }$$

$D_{1}$ represents the entrance diameter of the hole, $D_{2}$ represents the exit diameter of the hole, and *h* represents the thickness of the hole in SiC_f_/SiC CMC. Based on the tested results of LSCM, the taper angle could be calculated using the formula above.

## 3. Results and Discussion

### 3.1. Laser Pre-Drilling in Air of SiC_f_/SiC CMC

**Figure 3:**
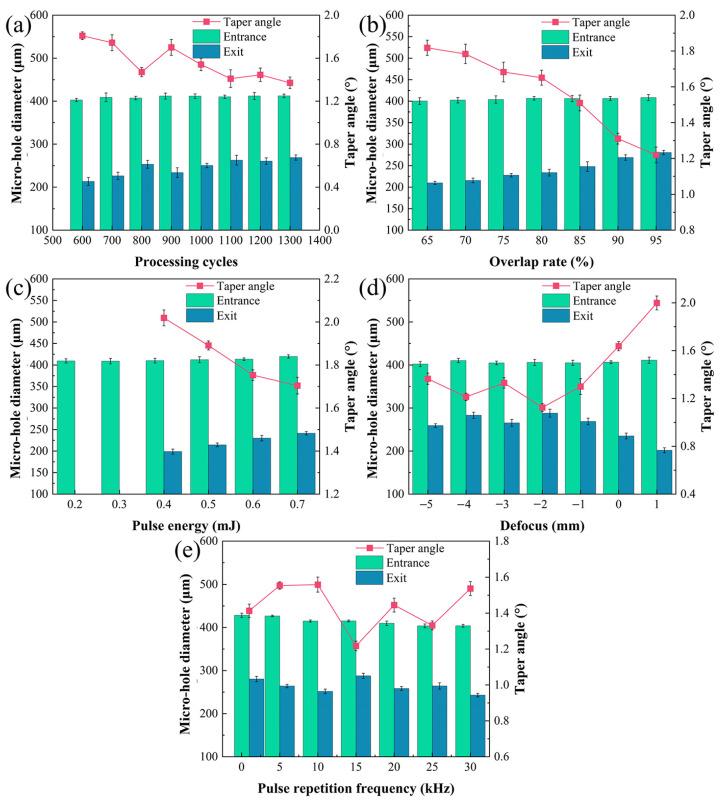
Statistical analyses of diameter and taper angle of micro-holes processed using laser pre-drilling in air with single-parameter variations: (**a**) processing cycles (fixed parameters of 80% overlap rate, 0.5 mJ single-pulse energy, −1 mm defocus, and 20 kHz pulse-repetition frequency); (**b**) overlap rate (fixed parameters of 900 processing cycles, 0.5 mJ single-pulse energy, −1 mm defocus, and 20 kHz pulse-repetition frequency); (**c**) single-pulse energy (fixed parameters of 900 processing cycles, 80% overlap rate, −1 mm defocus, and 20 kHz pulse-repetition frequency); (**d**) defocus (fixed parameters of 900 processing cycles, 80% overlap rate, 0.5 mJ single-pulse energy, and 20 kHz pulse-repetition frequency); (**e**) pulse-repetition frequency (fixed parameters of 900 processing cycles, 80% overlap rate, 0.5 mJ single-pulse energy, and −1 mm defocus).

The statistical analyses of diameter and taper angle of the micro-holes in the SiC_f_/SiC CMC processed with different single-parameter variations are shown in Figure 3. It can be seen that the diameters of the micro-hole entrances show almost no change for different laser processing, but the diameters of micro-hole exits show changes, which leads to the variation in taper angle. Comparatively, the regulation of overlap rate, defocus, and pulse-repetition frequency could exert a higher influence on the micro-hole dimension. With the increase in processing cycles, the diameter of the micro-hole exit increases gradually, but the taper angle of the micro-hole decreases gradually from 1.71° to 1.38°, as shown in Figure 3a. When the overlap rate rises, the diameter of the micro-hole exit increases continuously, which results in an obvious decrease in taper angle from 1.81° to 1.23°, as shown in Figure 3b. Interestingly, the basic laser parameters with a pulse energy lower than 0.4 mJ could not drill through the SiC_f_/SiC CMC. When the pulse energy is above 0.4 mJ, the diameter of the micro-hole exit increases continuously, and the taper angle decreases from 2.2° to 1.71°, as shown in Figure 3c. The influence of defocus on the micro-hole is diversified, as shown in Figure 3d. The diameter of the micro-hole exit increases, firstly reaching its minimum value at −2 mm, and then, the value obviously decreases, which produces a rapid rising in taper angle when the defocus is higher than −2 mm. Adjusting the pulse-repetition frequency obtains the highest diameter of the micro-hole entrance and exit, as shown in Figure 3e. With the increase in pulse-repetition frequency, the diameter of the micro-hole entrance decreases a little, while the diameter of the micro-hole exit decreases before 15 kHz and then increases sharply, following again by a decrease. As the pulse-repetition frequency increases, the diameter of the micro-hole entrance decreases slightly, while the diameter of the micro-hole exit first decreases, then increases sharply and subsequently decreases again. The pulse-repetition frequency of 15 kHz is an important turning point. Based on the single-parameter variation experiments, the optimal parameters for subsequent pre-drilling are 1100 processing cycles and 0.7 mJ pulse energy. The varying parameters are set as follows: an overlap rate ranging from 75% to 90%, a defocus from −4 mm to −1 mm, and a pulse-repetition frequency from 10 to 25 kHz.

According to the selected parameters, the optimized orthogonal experiments of laser pre-drilling in air were performed on the SiC_f_/SiC CMC. The detailed experimental parameters and results are given in Table 3. Considering the reverse effect of the overlap rate with defocus and pulse-repetition frequency, the parameter groups with varied overlap rate trends were set. The changes in micro-hole dimension and taper angle exhibit diversified variation with the parameters. When the pulse-repetition frequency is smaller than 20 kHz, the increase in the defocus and overlap rate mainly results in a rise in micro-hole size and a decline in taper angle. When the pulse-repetition frequency is 20 kHz, the higher overlap rate and defocus result in a bigger micro-hole exit diameter and a smaller taper angle. Once the pulse-repetition frequency increases to 25 kHz, the defocus plays a more important role. The higher the defocus, the larger the micro-hole exit diameter and the smaller the taper angle.

**Figure 4:**
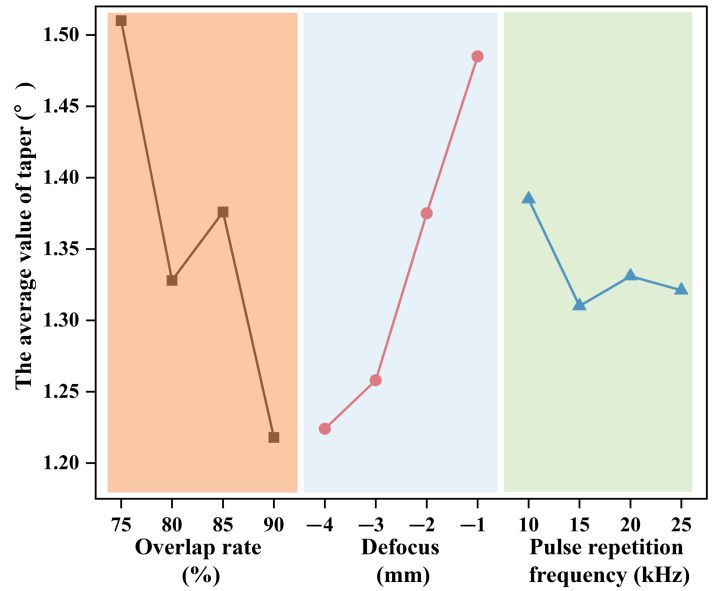
Variation in average micro-hole taper angle with pulse-repetition frequency, defocus, and overlap rate in laser pre-drilled SiC_f_/SiC CMC.

To further analyze the micro-hole shape, the taper angles obtained from laser pre-drilling in air-processed SiC_f_/SiC CMC, with varied single parameters, were equalized to obtain the average value. The variations in the average micro-hole taper angle of SiC_f_/SiC CMC with pulse-repetition frequency, defocus, and overlap rate are illustrated in Figure 4. The average taper angle generally decreased with the rise in pulse-repetition frequency, defocus, and overlap rate. In addition, there were some fluctuations in taper angle pertaining to the middle value of pulse-repetition frequency and overlap. This phenomenon indicates that the appropriate instantaneous energy density and laser spot shape play a determining role in the micro-hole shape. Generally, the increase in pulse-repetition frequency decreases the output energy amount, which is detrimental to laser ablation and the removal rate. Comparatively, an increased overlap rate and a higher defocus have an opposing effect. The increase in overlap rate enhances the absorbed energy in the irradiated region, while the increase in defocus reduces the absorbed energy. Thus, it is deduced that a higher specific laser energy ensures ablation, while a bigger laser spot benefits the removal of margin area. However, a higher overlap rate of laser spots requires more time. To balance the laser processing efficiency and quality, the optimal parameters of laser pre-drilling in air for the SiC_f_/SiC CMC are as follows: 1000 processing cycles, 0.7 mJ single-pulse energy, −4 mm defocus, 15 kHz pulse-repetition frequency, and 85% overlap rate. With the optimal laser processing parameters, the micro-hole with a diameter of about 340 µm in the SiC_f_/SiC CMC is processed using laser pre-drilling in air, and the time required is about 100 s.

**Figure 5:**
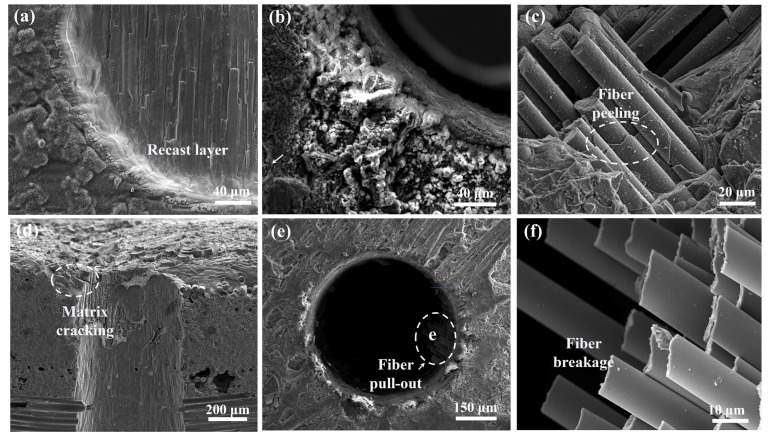
Typical morphology of micro-hole in SiC_f_/SiC CMC processed by laser pre-drilling in air: (**a**) recast layer on micro-hole entrance sidewall; (**b**) crystallization in entrance region; (**c**) debonding of SiC fiber and matrix; (**d**) cracking in entrance region; (**e**) residual SiC fiber at bottom of micro-hole; (**f**) breaking of SiC fibers.

To further study the effect of laser pre-drilling in air, the typical morphology of the micro-holes in SiC_f_/SiC CMC processed with optimal parameters were observed using SEM, and the results are shown in Figure 5. The observation on the blind micro-hole exhibits the recast layer in the entrance sidewall, indicating the remelting of SiC, as shown in Figure 5a. Clearly, the partly ablated SiC fibers are observed at the bottom of the micro-hole. Along the margin of the micro-hole, a sprout-like morphology is observed, which implies the occurrence of crystallization, as shown in Figure 5b. Further observation of the micro-hole wall exhibits the debonding of SiC fibers with the matrix, implying the interface stress caused by the thermal effect, as shown in Figure 5c. This phenomenon also demonstrates the relatively high thermal affected zone in the laser pre-drilled SiC_f_/SiC CMC. The profile of micro-hole exhibits a crack generated near the entrance, as shown in Figure 5d. This crack could be caused by the thermal stress generated by continuous heating. The observation of the micro-hole through SiC_f_/SiC CMC demonstrates residual SiC fibers in the region near the exit, as shown in Figure 5e. Further observation on the SiC fibers reveals that they are mostly broken, as shown in Figure 5f. This phenomenon could be ascribed to the enormous inner stress caused by the overheating at the last stage, which causes the random breaking of SiC fibers.

### 3.2. Laser Final-Drilling with Water Jet of SiC_f_/SiC CMC

**Figure 6:**
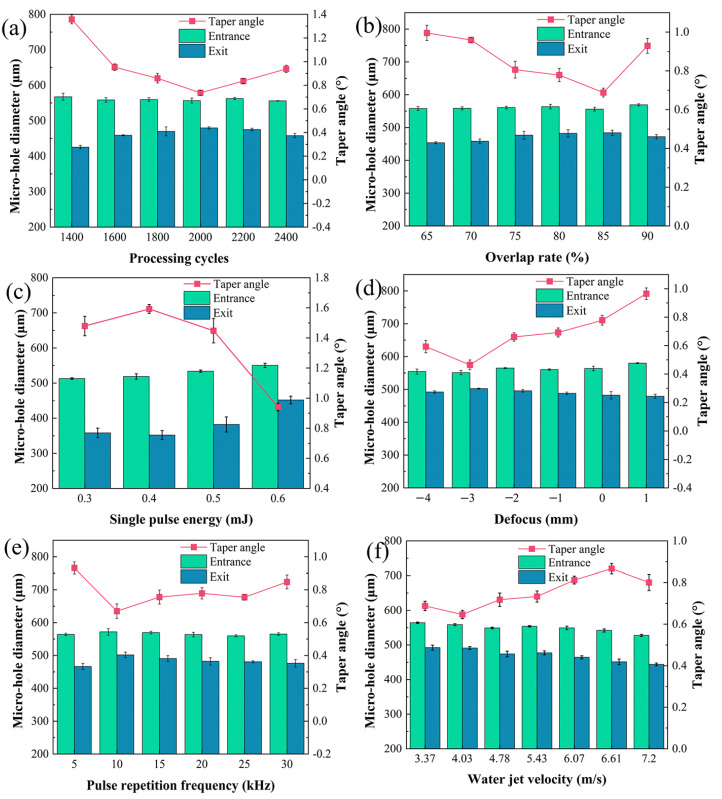
Statistical analyses of diameter and taper angle of micro-hole processed using laser final-drilling with water jet for single-parameter variations: (**a**) processing cycles (fixed parameters of 80% overlap rate, 0.6 mJ single-pulse energy, 0 mm defocus, 20 kHz pulse-repetition frequency, and 5.43 m/s waterjet velocity); (**b**) overlap rate (fixed parameters of 2000 processing cycles, 0.6 mJ single-pulse energy, 0 mm defocus, 20 kHz pulse-repetition frequency, and 5.43 m/s waterjet velocity); (**c**) single-pulse energy (fixed parameters of 2000 processing cycles, 80% overlap rate, 0 mm defocus, 20 kHz pulse-repetition frequency, and 5.43 m/s waterjet velocity); (**d**) defocus (fixed parameters of 2000 processing cycles, 80% overlap rate, 0.6 mJ single-pulse energy, 20 kHz pulse-repetition frequency, and 5.43 m/s waterjet velocity); (**e**) pulse-repetition frequency (fixed parameters of 2000 processing cycles, 80% overlap rate, 0.6 mJ single-pulse energy, 0 mm defocus, and 5.43 m/s waterjet velocity); (**f**) water jet velocity (fixed parameters of 2000 processing cycles, 80% overlap rate, 0.6 mJ single-pulse energy, 0 mm defocus, and 20 kHz pulse-repetition frequency).

Based on the laser pre-drilling in air experiments, the micro-holes in SiC_f_/SiC CMCs with an entrance of 340 μm are the specimens for laser final-drilling with a water jet. The statistical analyses of the diameter and taper angle of micro-holes in SiC_f_/SiC CMC processed with different single-parameter variations are shown in Figure 6. Compared with the diameter of the micro-hole entrance, the varied single-parameter experiments exert more influence on the diameter of the micro-hole exit, which leads to the diversified variation in micro-hole taper angle. With the increase in processing cycles, the diameter of the micro-hole entrance decreases a little, but the diameter of the micro-hole exit increases gradually at first and then decreases, as shown in Figure 6a. Correspondingly, the micro-hole taper angle declines from 1.38° to 0.76° with an increase in processing cycles up to 2000 cycles, and then, it increases to 0.95°. As shown in Figure 6b, the overlap rate has little effect on the micro-hole entrance diameter, except at 85%, where it decreases. However, the diameter of micro-hole exit almost increases with the increase in overlap rate, except at 90%. Therefore, the micro-hole taper angle decreases gradually from 0.99° to 0.71° when the overlap rate is lower than 85%, but it rises to 0.94° at an overlap rate of 90%. The increase in single-pulse energy slightly increases the diameter of the micro-hole entrance but significantly increases the diameter of the micro-hole exit, especially at 0.6 mJ, as shown in Figure 6c. Thus, the micro-hole taper angle slightly increases to 1.61° at first and then decreases to 0.92°. As the defocus changes from −4 mm to 1 mm, the diameter of micro-hole entrance mainly exhibits a gradual rising tendency, but the diameter of micro-hole exit mainly exhibits a gradual declining tendency, as shown in Figure 6d. The micro-hole taper angle decreases from 0.6° to 0.5° and then increases to 0.97°. With the increase in pulse-repetition frequency, the diameter of micro-hole entrance slightly changes, but the diameter of micro-hole exit increases firstly and then decreases, as shown in Figure 6e. Correspondingly, the micro-hole taper angle decreases from 0.94° to 0.68° and then increases to 0.85°. The increase in water jet velocity gradually decreases the diameters of micro-hole entrance and exit, but the decreasing rate of diameters of micro-hole entrance exit is higher, as shown in Figure 6f. Thus, the micro-hole taper angel increases from about 0.65° to 0.87° and then decreases to 0.81°. This phenomenon should be attributed to the splashing caused by a high water jet velocity, which results in laser refraction and decreases the removal of margin region. Based on the single-parameter variation experimental results of laser final-drilling with a water jet of SiC_f_/SiC CMC, the optimal parameters for subsequent final-drilling are 2000 processing cycles, 0.6 mJ single-pulse energy, and 4.03 m/s water jet velocity. The varying parameters are set as follows: the overlap rate ranging from 75% to 85%, the defocus from −4 mm to −2 mm, and the pulse-repetition frequency from 10 to 20 kHz.

According to the selected parameters, the optimized orthogonal experiments of laser final-drilling with a water jet were performed on the SiC_f_/SiC CMC. The detailed experimental parameters and results are given in Table 4. Due to the superimposed effect of overlap rate and defocus within the set parameter range, the pulse-repetition frequency is set with different change tendencies to explore their combined effect. It can be found that the changes in micro-hole dimension and taper angle exhibit diversified variations with the parameters. When the overlap rate is 80%, the increase in defocus and decrease in pulse-repetition frequency result in the continuous rise in micro-hole diameter but the reduction in micro-hole taper angle. As the overlap ratio is increased to 85%, the micro-hole diameter increases with the increase in defocus, but the variation of the micro-hole taper angle is disturbed by the disordered pulse repetition frequency, and their difference is quite small. When the overlap rate increases to 90%, the diameters of both the micro-hole entrance and exit, as well as the micro-hole taper angle, primarily increase with the rise in pulse-repetition frequency.

**Figure 7:**
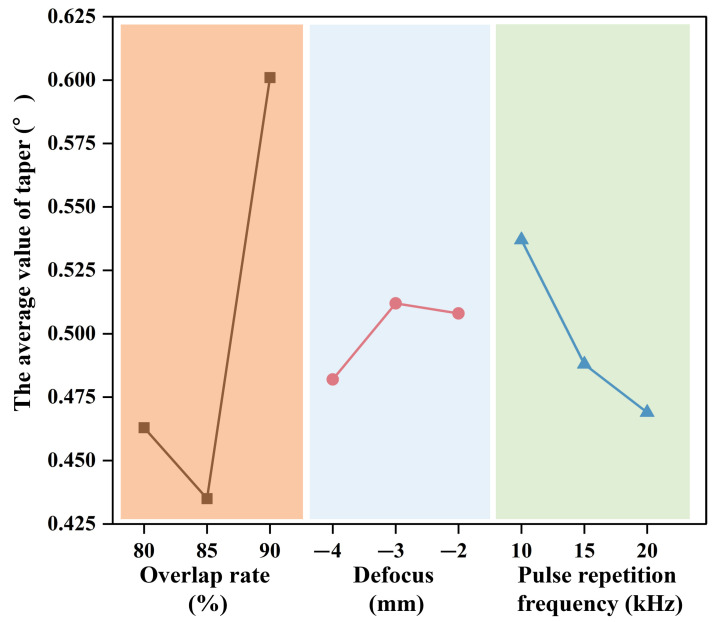
Variation in average micro-hole taper angle with pulse-repetition frequency, defocus, and overlap rate in laser final-drilled SiC_f_/SiC CMC.

The taper angles obtained from the laser final-drilling of water jet-processed SiC_f_/SiC CMC, with varied single parameters, were equalized to obtain the average value. The variations in the average micro-hole taper angle of SiC_f_/SiC CMC with pulse-repetition frequency, defocus, and overlap rate are illustrated in Figure 7. It is observed that the assistance of the water jet significantly decreases the taper angle. Comparatively, the influence of the overlap rate on the taper angle is higher than that of the defocus and pulse-repetition frequency. This phenomenon indicates the water jet could eliminate the dissipation of the laser energy to the adjacent region. In detail, the increase in overlap rate results in the decrease in taper angle from 0.47° to 0.43° and then an increase to 0.6°. The change in defocus from −4 mm to −1 mm results in the small rise in taper angle from 0.48° to 0.51°, and then, its value almost remains the same. On the other hand, the increase in pulse-repetition frequency causes the continuous decrease in taper angle from 0.53° to 0.47°. In summary, the balanced parameters of laser final-drilling with a water jet for SiC_f_/SiC CMC are as follows: 2000 processing cycles, 0.6 mJ single-pulse energy, −4 mm defocus, 10 kHz pulse-repetition frequency, 85% overlap rate, and 4.03 m/s water jet velocity. With the balanced laser processing parameters, the SiC_f_/SiC CMC with a pre-drilled micro-hole of about 340 µm is further processed using laser final-drilling with a water jet, and a final diameter of about 430 µm is obtained; the process takes about 96 s. Compared to laser pre-drilling in air, laser final-drilling with a water jet takes almost the same amount of time, but the material removal is only about half as much. That confirms that the water jet takes away the dissipated laser energy.

**Figure 8:**
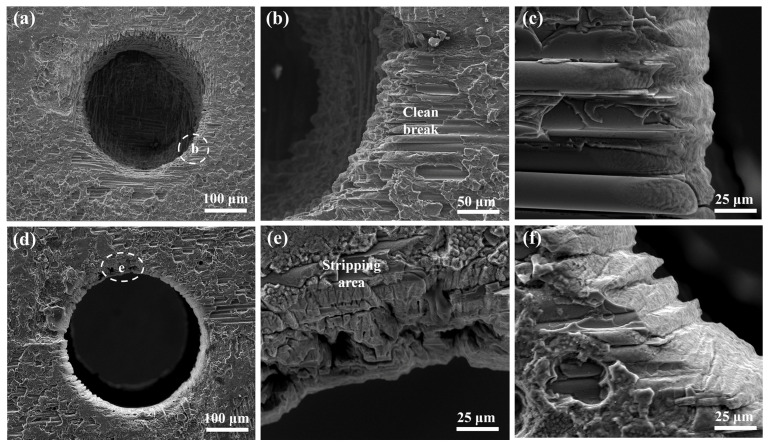
Typical morphology of micro-hole in SiC_f_/SiC CMC processed using laser final-drilling with water jet: (**a**) overall morphology of blind micro-hole; (**b**) broken SiC fiber on micro-hole entrance; (**c**) debonding of SiC fiber from matrix; (**d**) overall morphology through micro-hole; (**e**) stripped SiC fiber from composite; (**f**) surface morphology of composite ablated with laser.

The typical morphology of the micro-holes in the SiC_f_/SiC CMC processed using balanced parameters were observed with SEM, and the results are shown in Figure 8. Clearly, the assistance of the water jet obtains micro-holes with better shape and cleanliness. The blind micro-hole exhibits obvious exfoliated features in the entrance region, as shown in Figure 8a. There is no residual SiC fiber bunch in the micro-hole sidewall. A further observation of the entrance margin shows the exfoliated SiC matrix and broken SiC fibers, which can be attributed to the extreme inner stress caused by the intense alternating hot and cold temperatures, as shown in Figure 8b. The observation on the micro-hole sidewall also finds the debonding of the SiC fiber with the matrix, as shown in Figure 8c. Moreover, the laser-ablated SiC fiber surface and the micro-hole sidewall both exhibit the rough feature in small size. The observation made through the micro-hole reveals its relatively high roundness and smooth sidewall, as shown in Figure 8d. The edge of the entrance exhibits the stripping feature of the SiC fiber, implying the presence of high interfacial stress, as shown in Figure 8e. The detailed surface morphology of the micro-hole sidewall shows a zigzag feature that is almost consistent with the distribution of SiC fiber bunch, as shown in Figure 8f. This phenomenon also confirms the diverse physical properties of the SiC fiber and the SiC matrix, which result in the different removal efficiencies.

### 3.3. Influence of Medium and Laser Parameters on Laser Drilled Micro-Hole

**Figure 9:**
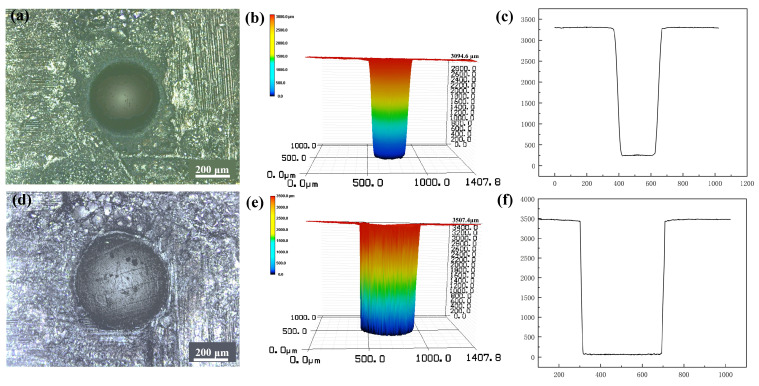
LSCM analyses of the typical micro-holes in SiC_f_/SiC CMC processed using laser pre-drilling in air (**a**–**c**) and laser final-drilling with a water jet (**d**–**f**): (**a**,**d**) surface morphology, (**b**,**e**) three-dimensional shape, (**c**,**f**) longitudinal cross-sectional profile.

According to the previous studies [40,41,42], laser ablation is mainly affected by the laser processing parameters and the processing environment. In fact, the laser processing parameters determine the input energy and its mode, while the processing environment determines the energy dissipation, chemical reaction during laser processing, and products formed on the processed interface. In this study, the laser pre-drilling in air makes full use of the laser energy to evaporate or remelt the SiC_f_/SiC CMC. Due to the low thermal conductivity and specific heat of the air, most of the laser energy is absorbed by SiC, which benefits ablation and material removal. On the contrary, water has much higher thermal conductivity and specific heat, which takes away most of the laser energy outside of the laser spot. Therefore, the laser pre-drilling in air has high processing efficiency, but the continuously absorbed laser heat results in thermal expansion and oxidation, which influences the processing precision and surface cleanliness. Comparatively, the laser final-drilling with a water jet has low processing efficiency, but its laser energy mainly ablates the region irradiated by the laser spot, which produces high processing precision. As shown in Figure 9a, the micro-hole in the SiC_f_/SiC CMC processed with laser pre-drilling in air has a relatively good roundness at the entrance, but the exit appears somewhat elliptical. Moreover, the entrance margin of the micro-hole has a relatively dark ring, indicating an oxidation zone [12]. The analyses of the micro-hole shape reveal an obvious taper, as shown in Figure 9b,c. The micro-hole processed using laser pre-drilling in air has an entrance diameter of 343 µm, an exit diameter of 218 µm, and a taper angle of 1.19°. The micro-hole processed using laser final-drilling with a water jet exhibits a relatively round entrance and exit, as shown in Figure 9d. There is no obvious oxidation zone in the entrance region. Though the taper angle still exists, the value shows an obvious decrease, as shown in Figure 9e,f. The micro-hole processed using laser final-drilling in air has an entrance diameter of 423 µm, an exit diameter of 385 µm, and a taper angle of 0.36°.

**Figure 10:**
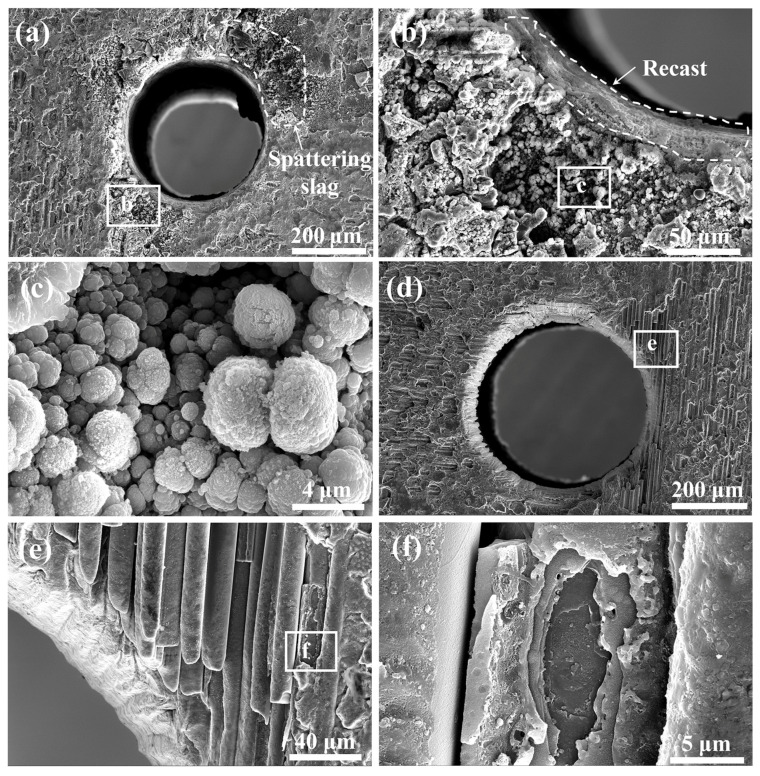
Detailed characterization of micro-hole in SiC_f_/SiC CMC processed using laser pre-drilling in air (**a**–**c**) and laser final-drilling with water jet (**d**–**f**): (**a**) morphology of entrance with spattering slag; (**b**) morphology of recast layer and spattering slag; (**c**) ultrafine spattering slag with cauliflower-like shape; (**d**) morphology of entrance with exfoliated SiC matrix; (**e**) partly ablated SiC fiber bunch; (**f**) morphology of SiC fiber with partly peeled transition layer.

Detailed observations of the micro-holes in the SiC_f_/SiC CMC processed using laser pre-drilling in air and laser final-drilling with a water jet demonstrate an obvious difference, as shown in Figure 10. The micro-hole processed using laser pre-drilling in air has a relatively good roundness, but its entrance has obvious spattering slag and cracks, as shown in Figure 10a. In the middle position of the micro-hole, a relatively big bulge is observed, which could be associated with oxides [47]. A further observation of the region with spattering slag reveals the fine particles formed inside, as shown in Figure 10b. Moreover, the recast layer is found in the sidewall of the micro-hole, which indicates the well-remelted inner layer of the micro-hole and a high thermal affected zone [48]. Fine particles in the spattering slag region exhibit a cauliflower-like shape and are connected with each other, as shown in Figure 10c. This morphology indicates that they are possibly the reaction products of spattered-SiC molten drops. Contrarily, the micro-hole processed using laser final-drilling with a water jet has better cleanliness and roundness, as shown in Figure 10d. Clearly, there is an obvious chamfer at the entrance edge, which could be attributed to the severe temperature change caused by the cracking of the micro-region [49]. In addition, the exfoliation of the SiC matrix is also observed in the entrance margin. A further observation of this region demonstrates the separated SiC fiber bunch without an envelope of the SiC matrix, as shown in Figure 10e. The SiC fibers have obvious features associated with laser ablation with gradually tapering ends, which also confirms the different laser ablation behaviors of the SiC fiber and matrix. The SiC fiber near the matrix exhibits the peeling of the transition layer on its surface, as shown in Figure 10f. Such a SiC fiber structure consumes more energy during laser ablation.

**Figure 11:**
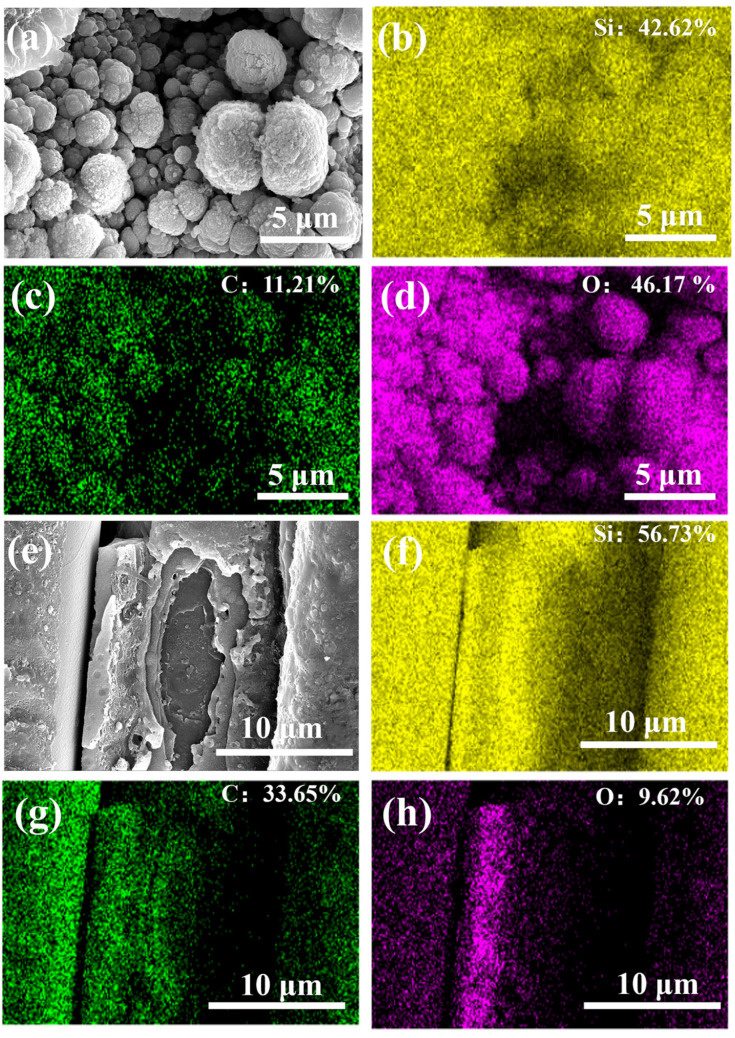
EDS analyses of cauliflower-like spattering slags (**a**–**d**) and SiC fiber with partly peeled transition layer (**e**–**h**) in laser-processed SiC_f_/SiC CMC: (**a**,**e**) overall morphology; (**b**,**f**) Si distribution; (**c**,**g**) C distribution; (**d**,**h**) O distribution.

The detailed EDS analyses of the cauliflower-like particles reveal that they are rich in Si and O, but there is still some C, as shown in Figure 11a–d. According to recent studies [50,51,52], these particles could be the oxides of SiC, possibly a mixture of SiC, SiO_2_, and SiO phases. SiC acts as the core of small particles, and its outer layer consists of SiO_2_ and SiO phases. The EDS analyses of the peeled SiC fiber reveal that it is mainly composed of Si and C, while the O content is significantly low, as shown in Figure 11e–h. In addition, O mainly distributes on the inner surface of the transition layer, which indicates that it may originate from the fabrication processing of SiC_f_/SiC CMC.

**Figure 12:**
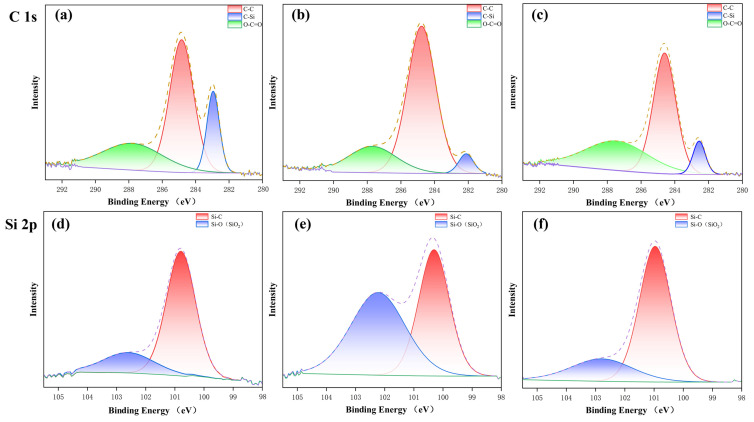
XPS analyses on initial SiC_f_/SiC CMC (**a**,**d**), laser pre-drilling in air-processed SiC_f_/SiC CMC (**b**,**e**), and laser final-drilling with water jet-processed SiC_f_/SiC CMC (**c**,**f**): (**a**–**c**) C 1s spectra, (**d**–**f**) Si 2p spectra.

To further investigate the chemical compound formed on the surface of laser-processed SiC_f_/SiC CMC, the XPS analyses were performed, and the spectra of C 1s and Si 2p are shown in Figure 12. For a comparative study, the initial SiC_f_/SiC CMC was also analyzed using XPS, as shown in Figure 12a,d. Its C 1s spectrum mainly consists of C-Si, C-C, and O-C=O peaks, and its Si 2p spectrum mainly consists of Si-C and Si-O. When combined with the peak strength, it is concluded that there is some SiO_2_ in the initial SiC_f_/SiC CMC, which may originate from its fabrication [53]. After the laser pre-drilling in air, the processed SiC_f_/SiC CMC exhibits an obviously weakened C-Si peak in the C 1s spectrum and a greatly strengthened Si-O peak in the Si 2p spectrum, as shown in Figure 12b,e. These results indicate the abundant formation of SiO_2_ on the SiC_f_/SiC surface processed with the laser pre-drilling in air, which further verifies the SEM observation. Due to the consumption of Si by oxidation, the C-Si peak is weakened, which promotes the slight strengthening of the C-C peak. After the laser final-drilling with a water jet, the processed SiC_f_/SiC CMC shows a slight increase in the C-Si peak in the C 1s spectrum, while the Si-O peak in the Si 2p spectrum significantly weakens, as shown in Figure 12c,f. These results imply the formation of SiO_2_ is quite limited, and SiC is the main phase of the SiC_f_/SiC surface processed using the laser final-drilling with a water jet. Therefore, the Si-O peak of the SiC_f_/SiC CMC processed using laser final-drilling with a water jet is almost similar to that of the initial SiC_f_/SiC CMC.

**Figure 13:**
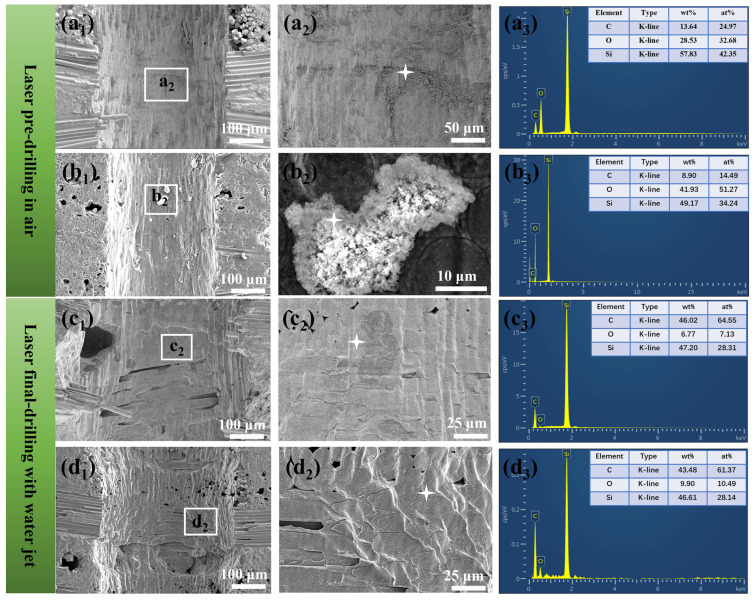
Typical SEM characterization on the micro-hole sidewall in SiC_f_/SiC CMC processed using laser pre-drilling in air and laser final-drilling with water jet: (**a_1_**,**b_1_**) overall morphology of the longitudinal section of th micro-hole processed by laser pre-drilling in air; (**a_2_**,**b_2_**) magnified images showing the morphology of agglomerates attached on the sidewall; (**a_3_**,**b_3_**) EDS analyses on the agglomerates; (**c_1_**,**d_1_**) overall morphology of the longitudinal section of the micro-hole processed using laser final-drilling with water jet; (**c_2_**,**d_2_**) differed surface roughness of the sidewall with different arranged SiC fibers; (**c_3_**,**d_3_**) EDS analyses on the sidewall (stars refer to the regions for EDS analyses).

In order to evaluate the quality of micro-holes in SiC_f_/SiC CMC processed using laser pre-drilling in air and laser final-drilling with a water jet, the morphology of the sidewall in the middle and bottom positions was observed, and the results are shown in Figure 13. Clearly, the middle sidewall surface of the micro-hole processed using laser pre-drilling in air mainly exhibits a wrinkled feature with some attached powders, as shown in Figure 13a_1_,a_2_. In fact, the feature of the SiC fiber is not observed in the sidewall. This morphology could be ascribed to the recast layer, which reconstructs the surface morphology. The EDS analysis of the powders reveals their high oxygen content, which indicates that they are oxide particles, as shown in Figure 13a_3_. The bottom sidewall of the micro-hole processed using laser pre-drilling in air has a similar wrinkled surface, but it has some large, attached particles, as shown in Figure 13b_1_,b_2_. The EDS analysis indicates that they are aggregated ultrafine oxides, as shown in Figure 13b_3_. Comparatively, the sidewall surface of the micro-hole processed using laser final-drilling with a water jet exhibits a different morphology. As shown in Figure 13c_1_,c_2_, the feature of SiC fibers is observed from the sidewall surface, even though its surface also exhibits a corrugated morphology. In the SiC fibers tangential to the sidewall surface, gaps are found inside, which could be ascribed to the partly stripped SiC fiber or matrix. Certainly, the inner stress induced by severe temperature changes also contributes to the stripping. The EDS analysis of the sidewall surface reveals its low oxygen content, as shown in Figure 13c_3_. The bottom sidewall of the micro-hole processed using laser final-drilling with a water jet also exhibits the corrugated morphology and fiber features, as shown in Figure 13d_1_,d_2_. However, the concave could be observed in the interface of intersected SiC fiber bunches, and the laser-ablated SiC fiber end exhibits a squamous morphology. This phenomenon implies the different ablation behavior of the SiC fiber and matrix.

According to previous studies [12,38,54], the laser ablation of Si_f_/SiC CMC is primarily determined by the absorbed energy, as vaporization and remelting are the main mechanisms for material removal. Moreover, the laser-induced plasma and reaction-formed SiO_2_ smoke also exert some influence on laser ablation, since they partly dissipate laser energy. Based on these factors, it can be concluded that the environment also exerts some influence on laser ablation. If the oxidation reaction is accelerated by the environment, more vaporized SiC is transformed into SiO_2_ smoke, which decreases the absorption rate of the laser energy and is detrimental to laser ablation. If the vaporized SiC and its oxides are removed rapidly, the absorption rate of the laser energy is increased, which benefits laser ablation. Certainly, the differences in density and thermal transmission behavior of the SiC fiber and matrix also affect the laser ablation behavior in the micro-region. Therefore, the surface quality and dimension precision of micro-hole processed using laser in the SiC_f_/SiC CMC are synergistically influenced by multiple factors.

**Figure 14:**
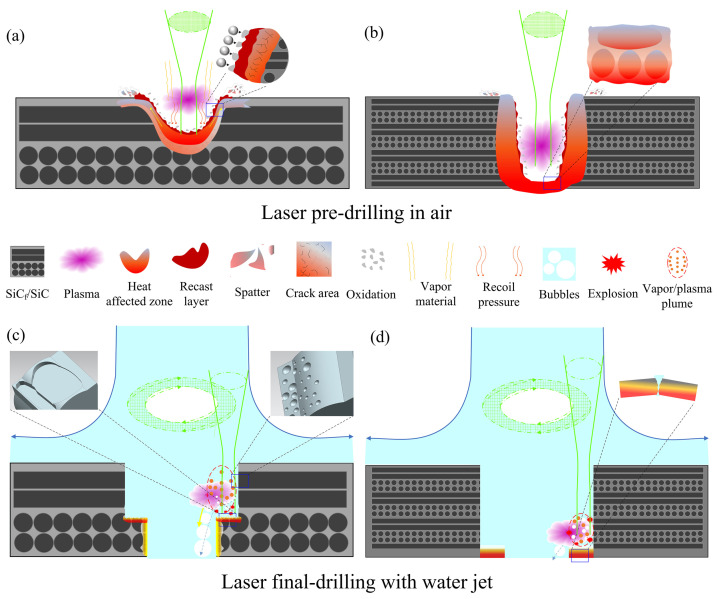
Schematic diagram illustrating the laser ablation mechanism of micro-holes in SiC_f_/SiC CMC processed using laser pre-drilling in air (**a**,**b**) and laser final-drilling with water jet (**c**,**d**).

Based on the above observations, the laser processing of SiC_f_/SiC CMC in different mediums is summarized, as shown in Figure 14. When the micro-holes of the SiC_f_/SiC CMC are processed using laser pre-drilling in air, there are two typical stages: the initial and final stage. At the initial stage, the nanosecond pulsed laser irradiation of the SiC_f_/SiC CMC results in rapid heating and remelting, due to the photothermal effect [48,54]. When the temperature of the irradiated region exceeds 2700 °C, the SiC fiber and SiC matrix are sublimated to form the SiC vapor, which produces the laser-induced plasma due to avalanche ionization, as shown in Figure 14a. Simultaneously, the presence of air promotes the oxidation of vaporized SiC, forming the SiO_2_ smoke. The aggregated SiO_2_ particles are scattered on the micro-hole sidewall or entrance. Moreover, the continuous heating leads to the overheating of the local SiC, where the inner vaporization produces the spattering of SiC melt. The spattering slags are attached to the sidewall or in the vicinity of the entrance and are oxidized subsequently. The oxidized spattering slags and attached SiO_2_ particles form the sidewall surface of the micro-hole. Due to the laser scanning route, a part of SiC vapor in the subsurface layer recrystallizes, producing a mixture in the local region. This evolution results in the microstructural degradation of the SiC_f_/SiC CMC, which is detrimental to its performance and decreases its service life. Certainly, the heat is transferred from the remelt layer to the adjacent position, forming a heat-affected zone with microcracks. With the progression of laser processing, remelting, vaporization, reaction, and spattering occur continuously, producing a rough sidewall surface in the micro-hole. When the micro-hole processing using laser pre-drilling in air is nearly finished, the final stage begins, as shown in Figure 14b. The continuous heating results in the thermal expansion of the bottom SiC_f_/SiC CMC, which promotes a slight deformation. Due to the deformation constraint, outside is the main expansion direction, which causes great inner stress [55]. Once the SiC_f_/SiC CMC is drilled through, the release of inner stress breaks the residual layer, which damages the local exit. In addition, with the deepening of the micro-hole, the spattering slags are difficult to expel outside and are gradually accumulated on the sidewall with subsequent oxidation, which continuously decreases the diameter [56,57]. Thus, the micro-hole processed using laser pre-drilling in air has a high taper angle.

The laser final-drilling with a water jet achieves a clean micro-hole in the SiC_f_/SiC CMC because of the scouring effect of water. As in the case of laser pre-drilling in air, the laser irradiation of the composite results in heating, remelting, and vaporization, but the presence of water produces bubbles and a steam explosion, due to the dramatic heating with a laser. As shown in Figure 14c, the water jet passing through the micro-hole removes a part of the vaporized SiC, but the residual SiC also generates the plasma above the laser-irradiated region. Even though the vaporized SiC reacts with water and is oxidized, it is removed by water [54]. The laser-produced heat is transferred to the inner layer, producing a subsurface-layer vapor, which produces spattering slags. They are eliminated by water as an oxidized SiC vapor [58]. The different densities and thermal transmission behaviors of the SiC fiber and matrix result in their diverse vaporization rates, which produce a rugged surface [59]. Furthermore, the steam explosion promotes the cavitation erosion effect, which accelerates the formation of a microgroove and pit morphology on the sidewall surface [60,61]. In addition, the diverse heat transmission behaviors of the SiC fiber and matrix produces relatively different temperature distributions in the same layer, resulting in inner stress. After the laser spot is removed, the rapid cooling effect of the water jet may amplify the inner stress, producing cracks in the local region. The steam explosion promotes the part exfoliation of SiC fiber and matrix, which produces micro-gaps [62]. With the progression of laser final-drilling with a water jet, the inner layer of the pre-drilled micro-hole is removed gradually. The microgrooves, pits, and micro-gaps are discontinuously formed on the micro-hole sidewall, which contributes to the main morphology of the inner surface. When the processing of the micro-hole using laser final-drilling with a water jet is nearly finished, the processing conditions are partly changed, as shown in Figure 14d. Since the thickness of the residual SiC_f_/SiC CMC is relatively small, the high stress generated by the laser heat produces cracks inside it, which may partly break the composite [55,62]. Due to this phenomenon, the micro-hole exit may contain SiC fibers with fractured features. In general, the presence of a water jet results in the dissipation in part of the laser energy, which decreases the laser processing efficiency. However, the laser final-drilling with a water jet effectively eliminates the oxides, when compared with the laser pre-drilling in air. Therefore, the micro-holes in the SiC_f_/SiC CMC processed using laser final-drilling with a water jet have a clean sidewall surface and a smaller taper angle. This two-step laser drilling simultaneously ensures high-quality processing (cleanliness and dimension precision) and high efficiency; thus, this method is applied in the processing of hard, brittle materials.

## 4. Conclusions

In this study, the nanosecond laser was utilized to process micro-holes in the SiC_f_/SiC CMC using the two-step drilling method. Specifically, the laser pre-drilling in air was applied to process the initial micro-holes with a rough surface, while the subsequent laser final-drilling with a water jet was used to process the inner layer, eliminating impurities and increasing the dimension precision. The surface morphology and shape of micro-holes in the SiC_f_/SiC CMC processed with different laser processing are characterized, and the main conclusions that are drawn are as follows:

(1) For the laser pre-drilling in air, its single-parameter variation experiments and optimized orthogonal experiments indicate that the optimal parameters for micro-hole processing in the SiC_f_/SiC CMC are 1000 processing cycles, 0.7 mJ single-pulse energy, −4 mm defocus, 15 kHz pulse-repetition frequency, and 85% overlap rate. With these parameters, the micro-hole with a diameter of about 340 µm in SiC_f_/SiC CMC is processed in about 100 s.

(2) For the laser final-drilling with a water jet, its single-parameter variation experiments and optimized orthogonal experiments indicate that the balanced parameters for micro-hole processing in the SiC_f_/SiC CMC are 2000 processing cycles, 0.6 mJ single-pulse energy, −4 mm defocus, 10 kHz pulse-repetition frequency, 85% overlap rate, and 4.03 m/s water jet velocity. With these parameters, the pre-drilled micro-hole with a diameter of about 340 µm in SiC_f_/SiC CMC is processed in about 96 s, and the final diameter of 423 µm is obtained.

(3) With the optimal parameters of laser pre-drilling in air, the micro-hole in the SiC_f_/SiC CMC with an entrance diameter of 343 μm, an exit diameter of 218 μm, and a taper angle of 1.19° is processed. Its entrance region contains spattering slags with oxidation, and its sidewall is covered by the recast layer, with a wrinkled morphology and some attached oxides, and the gradual increase in its recast amount results in a higher taper angle. This result could be attributed to the presence of oxygen, which promotes oxidation and increases processing efficiency.

(4) The laser final-drilling with a water jet efficiently decreases oxidation and eliminates the spattering slags, thus producing a micro-hole with a clean sidewall. However, the diverse densities and heat transfer behaviors of the SiC fiber and matrix result in the different ablations, which combine the steam explosion and cavitation erosion to form the microgroove sidewall morphology. Due to the effective elimination of spattering slags and oxides, the micro-hole in the SiC_f_/SiC CMC with an entrance diameter of 423 μm, an exit diameter of 385 μm, and a taper angle of 0.36° is processed, which indicates the high quality of micro-hole processing.

## Figures and Tables

**Table 1:** Single-parameter experiments of laser pre-drilling in air for SiC_f_/SiC CMC.

Parameters	Processing Cycles	Overlap Rate (%)	Pulse Energy (mJ)	Defocus (mm)	Pulse-Repetition Frequency (kHz)
Values	600, 700, 800, 900, 1000, 1100, 1200, 1300	65, 70, 75, 80, 85, 90, 95	0.2, 0.3, 0.4, 0.5, 0.6, 0.7	−5, −4, −3, −2, −1, 0, 1	0, 5, 10, 15, 20, 25, 0

**Table 2:** Single-parameter experiments of laser final-drilling with water jet for SiC_f_/SiC CMC.

Parameters	Processing Cycles	Overlap Rate (%)	Pulse Energy (mJ)	Defocus (mm)	Pulse-Repetition Frequency (kHz)	Water Jet Velocity (m/s)
Values	1400, 1600, 1800, 2000, 2200, 2400	65, 70, 75, 80, 85, 90	0.3, 0.4, 0.5, 0.6	−4, −3, −2, −1, 0, 1	5, 10, 15, 20, 25, 30	3.37, 4.03, 4.78, 5.43, 6.07, 6.61, 7.2

**Table 3:** Optimized orthogonal experiments of laser pre-drilling in air for SiC_f_/SiC CMC.

Experiment Number	Pulse-Repetition Frequency (kHz)	Defocus (mm)	Overlap Rate (%)	Micro-Hole Entrance Diameter (μm)	Micro-Hole Exit Diameter (μm)	Micro-Hole Taper Angle (°)
P1	10	−1	75	411.19	231.56	1.71
P2	10	−2	80	410.93	260.86	1.43
P3	10	−3	85	415.36	283.99	1.25
P4	10	−4	90	414.03	291.07	1.17
P5	15	−1	80	408.90	257.60	1.44
P6	15	−2	75	410.69	250.38	1.53
P7	15	−3	90	413.26	298.34	1.09
P8	15	−4	85	412.27	291.81	1.15
P9	20	−1	85	413.45	262.48	1.44
P10	20	−2	90	413.71	296.24	1.21
P11	20	−3	75	402.36	251.57	1.44
P12	20	−4	80	411.35	281.72	1.24
P13	25	−1	90	409.14	264.91	1.38
P14	25	−2	85	408.57	269.47	1.33
P15	25	−3	80	405.57	279.85	1.20
P16	25	−4	75	384.25	244.70	1.33

**Table 4:** Optimized orthogonal experiments of laser final-drilling with water jet for SiC_f_/SiC CMC.

Experiment Number	Overlap Rate (%)	Defocus (mm)	Pulse-Repetition Frequency (kHz)	Micro-Hole Entrance Diameter (μm)	Micro-Hole Exit Diameter (μm)	Micro-Hole Taper Angle (°)
F1	80	−2	20	532.91	475.76	0.55
F2	80	−3	15	552.32	505.47	0.45
F3	80	−4	10	563.78	521.88	0.40
F4	85	−2	15	545.52	501.66	0.42
F5	85	−3	10	558.53	511.14	0.45
F6	85	−2	20	561.69	515.56	0.44
F7	90	−2	10	556.63	497.88	0.56
F8	90	−3	20	565.25	499.42	0.63
F9	90	−4	15	564.21	500.41	0.61

## Data Availability

The original contributions presented in this study are included in the article. Further inquiries can be directed to the authors.
